# Antifungal Activity of *Plantago lanceolata* and *Sida ovata* Leaf Extracts against Dermatomycotic Fungi

**DOI:** 10.1155/2023/9957892

**Published:** 2023-08-05

**Authors:** Tamene Milkessa Jiru, Muluneh Getahun

**Affiliations:** ^1^Department of Environmental and Industrial Biotechnology, Institute of Biotechnology, University of Gondar, P.O. Box: 196, Gondar, Ethiopia; ^2^Department of Biotechnology, Institute of Biotechnology, University of Gondar, P.O. Box: 196, Gondar, Ethiopia

## Abstract

*Plantago lanceolata* and *Sida ovata* have been used as medicinal plants for centuries to cure numerous diseases. This study aimed to evaluate antifungal activity of *P. lanceolata* and *S. ovata* leaf extracts against dermatomycotic fungi. Crude extracts from leaves of both plants were prepared using methanol and ethyl acetate. Phytochemical screening of both plants leaves was performed. Antifungal activity of crude extracts was evaluated against three dermatomycotic fungi (*Candida albicans*, *Malassezia furfur*, and *Malassezia globosa*). In addition, minimum inhibitory concentration (MIC) of the extracts was determined by microbroth dilution method. Maximum inhibition zone of 32.00 ± 11.64 mm was exhibited when combined ethyl acetate extract of both plants was applied against *M. globosa*. Best effect of MIC was demonstrated by ethyl acetate extract against most tested dermatomycotic fungi. Average MIC of ethyl acetate and methanol extracts ranged as follows: (0.19 ± 0.00 to 0.65 ± 0.00 mg/mL and 0.19 ± 0.00 to 0.52 ± 0.22 mg/mL) and (0.65 ± 0.22 to 1.56 ± 0.00 mg/mL and 0.19 ± 0.00 to 0.52 ± 0.00 mg/mL), respectively. Their synergistic effect was better than the effect of individual plant leaf extract. Minimum fungicidal concentration (MFC) values varied across the fungal pathogens when extracts from both plants and their combinations were used. The findings from the current study support the traditional use of *P. lanceolata* and *S. ovata* against dermatomycotic fungal infections, which could potentially be exploited for the treatment of superficial infection in humans.

## 1. Introduction

Plants have been employed as sources of medicine in Ethiopia for thousands of years to treat different ailments. Studies suggest that about 80% of the Ethiopian population still depend on medicinal herbs for their basic healthcare system [1–3]. Even though traditional medicine and medicinal plants have a great role in the primary healthcare system in Ethiopia, the work that has been performed so far in the country to properly document and promote the associated knowledge concerning traditional medicine is scanty [4]. Therefore, documentation and promotion of traditional medicine and medicinal plants is considered necessary so that the knowledge and understanding on the extent and characteristics of traditional medicine can be preserved and utilized sustainably.

Because of their capability in providing many benefits to different socio-economic groups worldwide, especially in the line of modern medicine and pharmaceuticals, medicinal plants have gained great attention than ever. A wide range of plants parts such as leaves, roots, stems, bark, flowers, and seeds are used for therapeutic purposes as well as serve as precursors for the synthesis of various useful modern drugs due to their ethnomedical importance in nature. The medicinal potentials of these plants could be traceable to the bioactive phytochemical constituents that are responsible for the physiological action on the human body. Due to their versatility and multipurpose applications, plant-derived substances have gained attention recently. These plant-derived substances which enhance the usefulness of the plant are classified as phytochemicals [5].

*P. lanceolata* has been given a lot of attention due to its multiple therapeutic applications such as its wound healing remedy [6, 7]. This herbaceous plant was traditionally used in North Africa to treat wounds, burns, abscesses, inflammations, hemorrhoids, and fevers [8]. Previous reports have indicated that the plant was also effective against diarrhea, dysentery, antiviral, anti-inflammatory, astringent, antihelminthic, analgesic, analeptic, antihistaminic, antirheumatic, antitumor, antiulcer, diuretic, expectorant, and hypotensive in traditional medicine [8].

Among many popular medicinal plants, *P. lanceolata* and *S. ovata* have been reported to cure numerous diseases from cold to hepatitis, skin diseases, infectious diseases, problems concerning the digestive organs, respiratory organs, reproduction, the circulation, and for reducing fever [6].

*P. lanceolata* and *S. ovata* have been used either whole or crushed leaves, or juice from leaves of the two plants have been used to treat burns, stop bleeding, and treat all kinds of wounds to enhance the healing process [6].

As there are highly unexplored natural resources available in Ethiopia, focusing on searching for a novel, highly efficacious, better active, nontoxic, and affordable antifungal agents must be prior activity. In addition, there is scarcity of information about the synergistic antidermatomycotic activity leaf extracts of *P. lanceolata* and *S. ovata* in any part of the world including Ethiopia. Therefore, the current study aimed at evaluating the antimicrobial activity of *P. lanceolata* and *S. ovata* leaf extracts against dermatomycotic fungi that cause superficial infection in humans. The solvent extracts from the two plants were tested separately and synergistically.

## 2. Materials and Methods

### 2.1. Plant Materials Collection and Extraction

**Figure 1:**
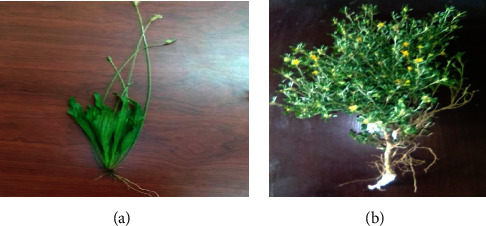
Image of (a) *P. lanceolata* and (b) *S. ovata* taken by Tamene Milkessa Jiru.

Leaves of *P. lanceolata* and *S. ovata* plants (Figure 1) were collected from University of Gondar compound, Northwest Ethiopia. Taxonomical identification and verification was performed at the department of biology by a Botanist in the University of Gondar.

The collected leaves were washed repeatedly and dried in an open air and protected from direct exposure to sun light to constant weight. Air dried leaves were grounded by analytical mill and packed in plastic bags.

Ethyl acetate and methanol solvents were used for extraction of *P. lanceolata* and *S. ovata* leaves, 30 g of dried and powdered of leaves of both plants were extracted with 300 mL of 99% ethyl acetate and 80% methanol in separate flasks and were shaked for about 7 days [9], then the obtained extracts were filtered using Whatman No. 1 filter paper and the solvents were evaporated using rotary distillation apparatus. In order to obtain a complete dry extract, the resultant extracts were transferred to glass dishes and left in 40°C oven for 24 h. The following extracts were obtained: methanolic extract (7 g) and ethyl acetate extract (11 g) from *P. lanceolata* leaf as well as methanolic extract (8 g) and ethyl acetate (9 g) from *S. ovata* leaf. Then, they were kept at 4°C until the assessments of their antidermatomycotic activities.

### 2.2. Qualitative Analysis of Phytochemicals

The presence of different phytochemical constituents of *P. lanceolata* and *S. ovata* leaves was detected using standard procedures. The presence of flavonoids, alkaloids, saponins tannins, and cardiac glycosides was characterized following the method of Adesegun et al. [10]. Steroids [11] and quinones [12] were also characterized. Furthermore, the presence of phenols, terpinoids, and carbohydrates was detected using the procedure of Martin et al. [13].

#### 2.2.1. Antidermatomycotic Test

The antidermatomycotic effect of the extracts was assessed using agar well diffusion assay [14]. The test pathogenic dermatomycotic fungi, namely, C*. albicans, M. furfur*, and *M. globosa* were obtained from microbiology unit, University of Gondar Comprehensive Specialized Hospital. These pathogenic isolates were first grown on sabouraud dextrose broth, incubated for 48 h. About 20 mL of sterilized SDA (g/L: glucose 40, peptone 10, and agar 15) medium was poured into Petri dishes. Suspension of the superficial pathogenic fungi was made in sterile normal saline and adjusted to the 0.5 MCFarland's standard. Small volume (20 *µ*L) of fungal suspension was added to each SDA plate and then evenly distributed by means of sterile swap over the agar surface. Agar wells were prepared using a sterilized cork borer with 6 mm diameter, 2 mm deep, and about 2.5 cm apart to minimize over lapping of zone. Dimethyl sulphoxide (DMSO) was used as negative control, while ketoconazole (KC) was used as positive control.

About 100 mg of dried leaf extract powder was resuspended in 1 mL of 10% DMSO. About 50 *µ*L of leaf extracts of both plants and ketoconazole or DMSO was placed into wells. Crude extracts and standard antibiotic were allowed to diffuse for about 40 min before incubation, and then the plates were incubated in an upright position at 30°C for 5 days. Results of the qualitative screening were recorded as the average diameter of the inhibition zone surrounding the wells containing the test solution. Results were compared with ketoconazole and DMSO.

Additionally, *P. lanceolata*–*S. ovata* leaf extract mixtures were prepared by mixing 25 *µ*L *P. lanceolata* leaf extract and 25 *µ*L *S. ovata* leaf extract that sum up 50 *µ*L, and similar procedures were followed for the remaining activities as it was performed for separate plant leaf extracts.

### 2.3. Determination of MIC and MFC

The MIC is the lowest concentration where no viability was observed after 24 h on the basis of metabolic activity. MIC value of each crude plant extract was estimated using broth microdilution method [15, 16]. 100 *μ*L of sabouraud dextrose broth was added to wells of a microtiter plate for each pathogenic fungal strain. A 100 *μ*L of each dissolved crude plant leaf extract (100 mg/mL) was then added in the first well and serially diluted row by row to give 50, 25, 12.5, 6.25, 3.125, 1.56, 0.78, 0.39, and 0.19 mg/mL, and 100 *μ*L of the mixture was discarded from the last row, thus leaving each diluted well with a volume of 100 *μ*L, except for the positive and negative controls. The test strains inocula were prepared from fresh 48 h grown cultures and were adjusted to McFarland standard, and 50 *μ*L was poured to the wells except the last well. Wells containing fungal suspensions and growth media, as well as DMSO, were used as negative control, and wells having fungal suspensions and growth media, as well as KC, were used as positive control. The wells were incubated for 72 h at 37°C temperature. Then, 20 *μ*L of resazurin reagent was added to each and every well to indicate respiratory activity, and a change in color from blue to pink was determined after incubating it for 72 h at 37°C temperature.

On the other hand, MFC was determined by first selecting the plate that showed no growth during MIC determination; a loopful from each plate subcultured onto SDA was incubated for further 48 h at 37°C. The lowest concentration at which no colonies were observed was noted as the MFC.

### 2.4. Data Analysis

The data collected through laboratory experiment were entered to Microsoft Excel 2013 and transferred to SPSS software version 20 for analysis. The results were expressed as mean value ± standard deviation (SD) of three replicates. Where applicable, the data were subjected to ANOVA, and differences between samples were determined by two-tailed *t*-test after Bonferroni error correction of the predictive value. *P* ≤ 0.05 was considered statistically significant.

## 3. Results

### 3.1. Phytochemical Detection

The different photochemical components of extracts of leaves of both plants were undertaken. All the tested phytochemical components, i.e., steroids, terpenoids, phenols, saponins, tannins, alkaloids, flavonoids, carbohydrates, quinines, and glycosides were found in *P. lanceolata* extract; however, steroids and tannins were not detected in *S. ovata* leaf extract.

### 3.2. Antidermatomycotic Fungal Activities

Test fungal pathogens used in the current study were *C. albicans*, *M. furfur*, and *M. globosa*. Both ethyl acetate and methanol extracts of *P. lanceolata* were assayed against these pathogens using agar well diffusion assay. The result in the current study revealed that the extracts from both plants, namely, *P. lanceolata* and *S. ovata* exhibit stronger inhibitory effect on *C. albicans*, *M. furfur*, and *M. globosa* in comparison to ketokonazle when applied separately and synergistically. Ethyl acetate extract showed higher effects to all tested dermatomycotic fungi. The maximum and minimum average zones of inhibition produced by ethyl acetate extract were 28.67 ± 6.69 mm and 18.17 ± 0.35 mm against *M. furfur* and *C. albicans*, respectively (Table 1). Similarly, the maximum and minimum average zones of inhibition produced by methanol extract were 21.33 ± 0.35 mm and 16.33 ± 1.40 mm against *M. furfur* and *C. albicans*, respectively (Table 1). Extracts from both solvents showed relatively substantial activities. The result of data analysis indicated that there was a significant antifungal effect difference between ethyl acetate and methanol extracts against *M. furfur* and *M. globosa*. Their effect was significantly different from ketoconazole (*p* ≤ 0.05) (Table 1). Statistically, no significantly different result was seen with both extracts against *C. albicans* positive control (*p* ≥ 0.05).

Concerning *S. ovata* leaf extract, *M. furfur* and *M. globosa* showed variable susceptibility to ethyl acetate and methanol extracts. There was relatively some degree of similar susceptibility observed by *C. albicans* to ethyl acetate extract (15.00 ± 0.58 mm) and methanol extract (15.00 ± 0.19 mm). The result obtained revealed that KC had better effect than both extracts. None of the extracts had better effect than KC when applied against *C. albicans* (21.00 ± 0.00 mm) and *M. furfur* (19.00 ± 0.00 mm) (Table 2). Statistically, no significant difference was seen with both extracts against *C. albicans* and *M. furfur*. However, their effect was significantly different against *M. globosa* (*p* ≥ 0.05) (Table 2).

As clearly shown in Table 3, better results were obtained from the combination of ethyl acetate and methanol crude extracts of both plants leaves against the tested microbes. *M. globosa* was highly susceptible to ethyl acetate extract with the average inhibition zone of 32.00 ± 11.64 mm. Statistically, no significance difference was seen with both extracts against all the tested superficial pathogenic fungi (*p* ≥ 0.05). But their effect was significantly different from Ketoconazole (*P* ≤ 0.05) (Table 4).

### 3.3. Determination of MIC and MFC

The MIC values of the tested extracts from the plant *P. lanceolata* leaf against tested pathogenic fungi were generally varied from pathogen to pathogen (Table 3). The ethyl acetate extract showed variable MIC value ranging from 0.19 ± 0.00 to 0.65 ± 0.00 mg/mL. The lowest MIC value was on *C. albicans* and *M. furfur*, and the highest MIC value was recorded on *M. globosa* (0.65 ± 0.00 mg/mL). Concerning the methanol extract, the lowest MIC value (0.19 ± 0.00 mg/mL) was recorded on both *M. furfur* and *M. globosa*, while the highest (0.52 ± 0.22 mg/mL) was observed against *C. albicans*.

The MIC values of the tested extracts from the plant *S. ovata* leaf against tested microbes generally varied from pathogen to pathogen. Generally, all tested fungal pathogens were inhibited by the lowest concentration of methanol extract compared to ethyl acetate extract. Therefore, methanol extract was a better choice of extract that can exert effect on tested microbes (Table 3).

Concerning the combined effects of the two plants leaves extracts; all the tested pathogens were inhibited by less concentration of ethyl acetate extracts of the two plants, i.e., 0.19 mg/mL. The same is true for methanol extract for both *M. furfur* and *M. globosa* with the exception of *C. albicans* that inhibited by 0.39 mg/mL.

Based on the result obtained, the MFC values of the tested extracts from the plant *P. lanceolata* were generally varied in the range from 0.19 to 3.12 mg/mL. Ethyl acetate and methanol were the most potent extracts with a MFC value of 0.19 mg/mL for *C. albicans* and *M. furfur*, respectively. Higher (3.12 mg/mL) MFC was recorded against *C. albicans* by methanol extract. The MFC values recorded by ethyl acetate and methanol were 1.56 ± 0.00 and 0.39 ± 0.00 mg/mL against *M. globosa*, respectively.

The MFC values of those extracts were from the plant *S. ovata* leaves varied across the fungal pathogens. The lowest MFC value of ethyl acetate, i.e., 3.12 ± 0.00 mg/mL, 1.56 ± 0.00 mg/mL, and 1.56 ± 0.00 mg/mL was recorded against *C. albicans*, *M. furfur*, and *M. globosa*, respectively, whereas the lowest MFC value of methanol extract, i.e., 1.56 ± 0.00 mm, 0.19 ± 0.00 mg/mL, and 0.39 ± 0.00 mg/mL was recorded against *C. albicans*, *M. furfur*, and *M. globosa*, respectively (Table 3).

The combined extracts from both plants showed considerable synergistic effects on *M. furfur*. But the best result was recorded by ethyl acetate extract at a concentration of 0.19 ± 0.00 mg/mL against *M. furfur* and *M. globosa.* On the other hand, *C. albicans*, *M. furfur*, and *M. globosa* could be killed by methanol extract at the concentrations of 0.52 ± 0.00 mg/mL, 0.19 ± 0.00 mg/mL, and 0.39 ± 0.00 mg/mL, respectively (Table 3). In general, varied MIC and MFC values were obtained; however, significantly better yields were obtained when the combined extracts act up on the dermatomycotic fungi.

## 4. Discussion

In the current study, qualitative phytochemical analysis of both *P. lanceolata* and *S. ovata* leaf extracts was undertaken. *P. lanceolata* leaf crude extract found to contain steroids, terpenoids, phenols, saponins, tannins, alkaloids, flavonoids, carbohydrates, quinones, and glycosides. The above finding was in line with the work peformed by Fayera et al. [8], who confirmed secondary metabolites belonging to different phytochemical groups from this plant including saponins, tannins, alkaloids, terpenoids, flavonoids, and phenolic compounds. Likewise, crude extracts of *S. ovata* leaf were found to contain terpenoids, phenols, saponins, alkaloids, flavonoids, carbohydrates, quinones, and glycosides. The above finding was in line with the research report by Irobi et al. [17] whose analysis of this plant showed the presence of carbohydrates, alkaloids, saponins, fixed oil, and phytosterols. The above finding was still in concurrent with the study report carried out by Souza et al. [18] whose analysis of *Spiranthera odoratissima* showed the presence of organic acids, reducing sugars, flavonoids, saponin compounds, coumarin compounds, phenolics, tannins, purine compounds, catechins, flavonol derivatives, sesquiterpene lactones, and anthraquinones. This shows that the phytochemicals from the studied plants leaves extracts had contributed to their antimicrobial activity against the tested clinically isolated dermatomycotic fungi. Studies suggest that phenolic compounds disrupt microbial cell membranes [19] by changing the permeability and causing the leakage of cellular content or interfere with membrane proteins resulting in structure disrupting [20–23]. Flavonoids also affect the cell membranes of microorganisms and inhibit nucleic acid synthesis (caused by topoisomerase inhibition) and energy metabolism (caused by NADH-cytochrome c reductase or ATP synthase inhibition) [24, 25]. In addition, this secondary metabolite interrupts cell wall and cell membrane synthesis. On the other hand, alkaloids possess the ability to disrupt the cell wall, inhibit DNA synthesis, and interrupt activity of enzymes (esterase, DNA-polymerase, and RNA-polymerase) or cell respiration [26]. Quinones have a potential to form irreversible complex with nucleophilic amino acids in proteins. Probable targets in the microbial cell are surface-exposed adhesins, cell wall polypeptides, and membrane-bound enzymes [21]. Tannins may act by inactivating/inhibiting microbial adhesins, enzymes, and cell envelope transport proteins [27]. Tannins also affect microorganisms in a number of ways which include destabilizing their cell membrane, disrupting their metabolism, as well as depriving the substrates required for microbial growth [22].

The three tested human pathogenic dermatomycotic fungi showed different antimicrobial susceptibility to leaf extracts of both plants. The possible reason for this difference in antifungal activity could be the susceptibility pattern of the pathogens and the differences in mechanism of actions of the metabolites in each solvent fraction.

In this study, all the crude extracts were applied against three dermatomycotic (superficial) fungi. So, the antifungal activity of ethyl acetate and methanol extracts from the plant *P. lanceoleta* was tested against three fungal pathogens. It was found that the plant had antifungal activity against those microbes. This finding was in accordance with the studies conducted in Ethiopia by Fayera et al. [8].

Previous studies on evaluating antimicrobial activities of the two medicinal plants against dermatomycotic fungi are scanty; the results cannot be compared with any other studies. However, we have tried to compare the results against the works performed on pathogenic bacteria and fungi.

The antimicrobial activity of *P. lanceolata* in this study is concurrent with the findings of a study by Eshetie et al. [28]. Likewise, the plant *S. ovata* showed different antifungal activities to the three tested dermatomycotic fungal pathogens. The possible explanation for this is that the plants are rich in compounds with potential antifungal activities.

Ethyl acetate extract from *P. lanceolata* showed higher effects to all tested fungi compared to methanol extract with the average zones of inhibition 28.67 ± 6.69 mm, 27.00 ± 1.00 mm, and 18.17 ± 0.35 mm against *M. furfur*, *M. globosa*, and *C. albicans*, respectively. The average zones of inhibition formed by ethyl acetate were better than the report from Eshetie et al. [28]. This difference could be due to methodology difference, the age of the plant, and the difference in the defense mechanism against foreign chemicals by bacterial and fungal species.

The current study indicated that both ethyl acetate and methanol extracts of the plant *S. ovata* leaf had good antifungal effect on *C. albicans*, *M. furfur*, and *M. globosa*. Better result was obtained from the study report by Akilandeswariet et al. [29] on antimicrobial activity of leaf extracts of *S. acuta* Burm related species. On the other hand, Abdulrahman et al. [30] obtained poor result on the same plant leaf extract. The variation could be differences in ways of extract preparation, the difference in the species of plants, the different geographical and environmental conditions during the growth of the plant as well as the difference in the age of the plants.

According to the findings of this study, the synergistic effect *P. lanceolata* and *S. ovata* leaf extracts had larger inhibition zone against tested dermatomycotic fungal pathogens than that of the leave extract of each plant applied separately. The synergistic effect may be attributed to the possibilities of the extracts affecting the different fungi processes in the cell [31]. This might be the basic reason why the local community widely uses the mixture of crude extracts from those plants to treat different pathogenic fungal infections.

The present study revealed that the lowest MIC values observed by ethyl acetate extract from the *P. lanceolata* leaf against *M. furfur* and *C. albicans* were 0.19 ± 0.00, while the MIC value of the same extract against *M. globosa* was 0.65 ± 0.00. The MIC value recorded in this study was much better than the finding report by Eshetie et al. [28] who reported the MIC value of 12.5 mg/mL–50 mg/mL against different tested bacteria. This difference could be due to experimental pathogens utilized. The plant could be effective for fungal pathogens compared to bacterial pathogens.

Concerning *S. ovata* leaf extracts, all the tested fungal pathogens were inhibited by the lowest concentration of methanol extract compared to ethyl acetate extract. Therefore, methanol extract was a better choice of extract that can exert effect on the tested microbes (Table 3).

Concerning the MIC value of combined effects of the extracts, the two extracts proven to have an increased efficacy (lowest concentration of 0.19 mg/mL) when compared to individual application of extracts (Table 3). The same is true for methanol extract for both *M. furfur* and *M. globosa* with the exception of *C. albicans* that inhibited by 0.39 mg/mL (Table 3).

## 5. Conclusion

From the overall study, it can be concluded that extracts from *P. lanceolata* and *S. ovata* leaf were found to contain various phytochemical secondary metabolites. The extracts from both plants showed antimicrobial activity when used against the superficial human fungal pathogens. The combined effect of crude extracts from both plants leaves was found to have higher antifungal activity than the effect of individual plant leaf extracts. The findings from the current study support the traditional use of *P. lanceolata* and *S. ovata* against dermatomycotic fungal infections, which could potentially be exploited for the treatment of superficial infection in humans.

## Figures and Tables

**Table 1:** Antifungal activities of *P. lanceolata* leaf extract (mean ± SD, mm).

Crude extracts	*C. albicans*	*M. furfur*	*M. globosa*
Ethyl acetate extract	18.17 ± 0.35^b^	28.67 ± 6.69^a^	27.00 ± 1.00^a^
Methanol extract	16.33 ± 1.40^b^	21.33 ± 0.35^b^	20.33 ± 1.18^b^
KC	21.00 ± 0.00^a^	19.00 ± 0.00^c^	15.00 ± 0.00^c^
DMSO	0.00 ± 0.00^c^	0.00 ± 0.00^c^	0.00 ± 0.00^d^

Values described by the different letters within the same column are significantly different (*P* ≤ 0.05). DMSO: dimethyl sulphoxide and KC-: ketoconazole.

**Table 2:** Antifungal activities of *S. ovata* leaf extract (mean ± SD, mm).

Crude extracts	*C. albicans*	*M. furfur*	*M. globosa*
Ethyl acetate	15.00 ± 0.58^b^	17.33 ± 1.71^b^	18.00 ± 0.19^a^
Methanol	15.00 ± 0.19^b^	16.00 ± 1.69^b^	15.33 ± 2.18^b^
KC	21.00 ± 0.00^a^	19.00 ± 0.00^a^	15.00 ± 0.00^b^
DMSO	0.00 ± 0.00^c^	0.00 ± 0.00^c^	0.00 ± 0.00^c^

Values described by the different letters within the same column are significantly different (*P* ≤ 0.05).

**Table 3:** MIC (mg/mL) and MFC (mg/mL) value of each extract of *P. lanceolata* and *S. ovata* leaves and their combined effect against dermatomycotic fungi.

	Plants	Crude extracts	Dermatomycoytic fungi		
			*C. albicans*	*M. furfur*	*M. globosa*
MIC	*P. lanceolata*	Ethyl acetate	0.19 ± 0.00	0.19 ± 0.00	0.65 ± 0.00
		Methanol	0.52 ± 0.22	0.19 ± 0.00	0.19 ± 0.00
	*S. ovata*	Ethyl acetate	1.56 ± 0.00	0.65 ± 0.22	0.78 ± 0.00
		Methanol	0.19 ± 0.00	0.52 ± 0.00	0.19 ± 0.00
	Combined effect	Ethyl acetate	0.19 ± 0.00	0.19 ± 0.22	0.19 ± 0.00
		Methanol	0.39 ± 0.00	0.19 ± 0.00	0.19 ± 0.00

MFC	*P. lanceolata*	Ethyl acetate	0.19 ± 0.00	0.78 ± 0.00	1.56 ± 0.00
		Methanol	3.12 ± 0.00	0.19 ± 0.00	0.39 ± 0.00
	*S. ovata*	Ethyl acetate	3.12 ± 0.00	1.56 ± 0.00	1.56 ± 0.00
		Methanol	1.56 ± 0.00	0.19 ± 0.0	0.39 ± 0.00
	Combined effect	Ethyl acetate	0.39 ± 0.00	0.19 ± 0.00	0.19 ± 0.00
		Methanol	0.52 ± 0.00	0.19 ± 0.0	0.39 ± 0.00

Average MIC and MFC values were expressed as mean ± standard deviation (*n* = 3), and analysis was performed with one-way ANOVA followed by the Bonferroni test.

**Table 4:** Combined effect of *P. lanceolata* and *S. ovata* leaf extract (mean ± SD, mm).

Crude extracts	*C. albicans*	*M. furfur*	*M. globosa*
Ethyl acetate	25.67 ± 2.08^a^	28.33 ± 0.62^a^	32.00 ± 11.64^a^
Methanol	24.67 ± 4.04^a^	26.00 ± 0.41^a^	27.00 ± 0.41^a^
KC	21.00 ± 0.00^b^	19.00 ± 0.00^b^	15.00 ± 0.00^b^
DMSO	0.00 ± 0.00^c^	0.00 ± 0.00^c^	0.00 ± 0.00^c^

Values described by the different letters within the same column are significantly different (*P* ≤ 0.05).

## Data Availability

The data used in this study are included in the article.
